# Transactions of the American Dental Association

**Published:** 1890-02

**Authors:** 


TRANSACTIONS OF THE AMERICAN DENTAL ASSOCIATION at the Twenty-ninth Annual Session, held at Saratoga Springs, N. Y., August, 1889. Philadelphia: S. S. White Dental Manufacturing Co., Publishers.

This report contains the proceedings and reports of the various standing committees and the essays read before the association, the whole making a very interesting volume.

